# Associations of Cerebral Small Vessel Disease and Chronic Kidney Disease in Patients With Acute Intracerebral Hemorrhage: A Cross-Sectional Study

**DOI:** 10.1212/WNL.0000000000209540

**Published:** 2024-06-18

**Authors:** Philip S. Nash, Simon Fandler-Höfler, Gareth Ambler, Wenpeng Zhang, Hatice Ozkan, Martina Locatelli, Yang Du, Lena Obergottsberger, Gerit Wünsch, Hans Rolf Jäger, Christian Enzinger, David C. Wheeler, Robert J. Simister, Thomas Gattringer, David J. Werring

**Affiliations:** From the UCL Stroke Research Centre (P.S.N., S.F.-H., W.Z., H.O., M.L., Y.D., R.J.S., D.J.W.), Department of Brain Repair and Rehabilitation, and Comprehensive Stroke Service (P.S.N., H.O., R.J.S., D.J.W.), National Hospital for Neurology and Neurosurgery, University College London Hospitals NHS Trust, UCL Queen Square Institute of Neurology, London, United Kingdom; Department of Neurology (S.F.-H., L.O., C.E., T.G.), Medical University of Graz, Austria; Department of Statistical Science (G.A.), University College London, United Kingdom; Institute for Medical Informatics (G.W.), Statistics and Documentation, Medical University of Graz, Austria; Lysholm Department of Neuroradiology and the Neuroradiological Academic Unit (H.R.J.), Department of Brain Repair and Rehabilitation, UCL Institute of Neurology; Department of Renal Medicine (D.C.W.), University College London, United Kingdom; and Division of Neuroradiology (T.G.), Vascular and Interventional Radiology, Department of Radiology, Medical University of Graz, Austria.

## Abstract

**Background and Objectives:**

Chronic kidney disease (CKD) may be associated with the pathogenesis and phenotype of cerebral small vessel disease (SVD), which is the commonest cause of intracerebral hemorrhage (ICH). The purpose of this study was to investigate the associations of CKD with ICH neuroimaging phenotype, volume, and location, total burden of small vessel disease, and its individual components.

**Methods:**

In 2 cohorts of consecutive patients with ICH evaluated with MRI, we investigated the frequency and severity of CKD based on established Kidney Disease Improving Global Outcomes criteria, requiring estimated glomerular filtration rate (eGFR) measurements <60 mL/min/1.73^2^ ≥ 3 months apart to define CKD. MRI scans were rated for ICH neuroimaging phenotype (arteriolosclerosis, cerebral amyloid angiopathy, mixed location SVD, or cryptogenic ICH) and the presence of markers of SVD (white matter hyperintensities [WMHs], cerebral microbleeds [CMBs], lacunes, and enlarged perivascular spaces, defined according to the STandards for ReportIng Vascular changes on nEuroimaging criteria). We used multinomial, binomial logistic, and ordinal logistic regression models adjusted for age, sex, hypertension, and diabetes to account for possible confounding caused by shared risk factors of CKD and SVD.

**Results:**

Of 875 patients (mean age 66 years, 42% female), 146 (16.7%) had CKD. After adjusting for age, sex, and comorbidities, patients with CKD had higher rates of mixed SVD than those with eGFR >60 (relative risk ratio 2.39, 95% CI 1.16–4.94, *p* = 0.019). Severe WMHs, deep microbleeds, and lacunes were more frequent in patients with CKD, as was a higher overall SVD burden score (odds ratio 1.83 for each point on the ordinal scale, 95% CI 1.31–2.56, *p* < 0.001). Patients with eGFR ≤30 had more CMBs (median 7 [interquartile range 1–23] vs 2 [0–8] for those with eGFR >30, *p* = 0.007).

**Discussion:**

In patients with ICH, CKD was associated with SVD burden, a mixed SVD phenotype, and markers of arteriolosclerosis. Our findings indicate that CKD might independently contribute to the pathogenesis of arteriolosclerosis and mixed SVD, although we could not definitively account for the severity of shared risk factors. Longitudinal and experimental studies are, therefore, needed to investigate causal associations. Nevertheless, stroke clinicians should be aware of CKD as a potentially independent and modifiable risk factor of SVD.

## Introduction

Intracerebral hemorrhage (ICH) has high rates of mortality and survivor disability.^1^ Unlike ischemic stroke, the incidence and prognosis of ICH have not substantially improved despite better hypertension screening and treatment.^2,3^ Approximately 80% of nontraumatic ICH is caused by cerebral small vessel diseases (SVDs) including arteriolosclerosis (also termed deep perforator arteriopathy or hypertensive arteriopathy) and cerebral amyloid angiopathy (CAA). Neuroimaging markers of small vessel injury may be hemorrhagic (e.g., cerebral microbleeds [CMBs]) or nonhemorrhagic (e.g., lacunes or white matter hyperintensities [WMHs]) and are best observed on brain MRI including sequences sensitive to paramagnetic susceptibility effects.^4^ A “mixed SVD” pattern may be caused either by coexistence of arteriolosclerosis and CAA or by severe arteriolosclerosis alone^5^; a recent histopathologic study provides evidence that at least some lobar microbleeds are caused by arteriolosclerosis.^6^ Both CAA and arteriolosclerosis are strongly associated with age, and arteriolosclerosis is strongly associated with vascular risk factors, particularly hypertension.

Chronic kidney disease (CKD) is an independent risk factor of ICH^7^ and is associated with SVD, particularly in community-based populations, such as those with hypertension or diabetes.^8^ However, associations of SVD and CKD in ICH populations have not been well characterized, yet are relevant for understanding the potential role of CKD in pathogenesis and thus prevention. Our recent study found a higher burden of SVD in patients with acute ICH and reduced estimated glomerular filtration rate (eGFR) on admission, but was limited by access to only brain CT, not allowing a complete classification of SVD subtypes, and only measuring renal function at a single time point.^9^

Indeed, most studies investigating CKD in stroke populations have used a single laboratory measurement indicating “renal impairment” rather than the internationally recommended Kidney Disease International: Improving Global Outcomes (KDIGO) definition of CKD: confirmed eGFR <60 mL/min/1.73^2^ and/or albuminuria >30 mg/g on at least 2 occasions ≥3 months apart.^10^ Using a single measurement will overestimate the true prevalence of CKD because it does not account for acute kidney injury, which is common in acute stroke (e.g., a prevalence of 24.5% in a stroke thrombectomy population^11^).

We, therefore, performed a detailed cross-sectional analysis of a large, unselected, 2-center hospital ICH population, describing the clinical and neuroimaging phenotypes associated with CKD, using brain MRI to classify SVD and the KDIGO-validated definition for CKD assessed by eGFR. We investigated associations between CKD and (1) ICH neuroimaging phenotype (arteriolosclerosis, CAA, mixed-location SVD, and “cryptogenic” ICH with no evidence of SVD); (2) ICH volume and location; (3) total SVD burden (based on a widely used ordinal score^12^); and (4) individual SVD markers. We hypothesized that CKD would be common in all patients with ICH caused by SVD, associated with the overall burden of SVD, most common in ICH associated with arteriolosclerosis and uncommon in cryptogenic (i.e., unexplained) ICH.

## Methods

### Patient Selection

For this cross-sectional study, we included individual patient data collected from 2 large consecutive cohorts of patients with acute ICH. The Stroke Investigation Group in North and Central London (SIGNAL) registry enrolled consecutive patients older than 18 years with imaging-confirmed acute ischemic stroke or ICH from the North Central London region (population approximately 1.5 million) treated at the University College London (UCL) Hospitals NHS Foundation Trust from 2015 to 2021.^13^ The SIGNAL registry included detailed data on clinical and neuroimaging phenotypes collected using standardized data collection sheets. Hypertension was defined as either previous diagnosis or preexisting use of antihypertensive medication. Diabetes was defined as ongoing or newly initiated therapy with antidiabetic drugs or hemoglobin A1c of ≥6.5%. Neuroimaging with MRI was the standard of care for all patients unless contraindicated.

The Graz ICH cohort study has been described previously^14^; in brief, it retrospectively identified all consecutive adult patients presenting to the University Hospital of Graz with first-ever ICH, between 2008 and 2021, and collected all baseline data using standardized data collection sheets. Similar to the SIGNAL registry, neuroimaging with MRI was the standard of care for all patients unless contraindicated.

For this analysis, we screened all patients with ICH from these 2 cohorts for eligibility. We excluded all patients who did not have an MRI of diagnostic quality; had a structural, macrovascular, or other secondary (non-SVD–related) cause of ICH; had intraventricular hemorrhage only; or did not have renal data available. Because the 2 studies recruited over different time periods, we compared the baseline characteristics according to study center to ensure that combining the cohorts was valid.

### Data Assessment

We electronically extracted renal biochemistry profiles from the respective hospital electronic health records at the following time points: admission, 48 hours after admission, days 6–8 after admission, hospital discharge, most recent test ≥3 months before ICH, and most recent test ≥3 months after ICH.

For those with eGFR <60 mL/min per 1.73^2^, CKD diagnoses were validated, according to KDIGO criteria,^10^ by a trained nephrologist (P.S.N.) using the abovementioned renal data. If necessary, we reviewed the primary care electronic health record (for UCL Hospitals patients) or the electronic patient records (for Graz patients). Participants who had acute kidney injury on admission were only included in the CKD group if the diagnosis of CKD could be validated. We did not have systematically collected data available on albuminuria.

### Neuroimaging Analysis

MRI protocols included at least the following sequences: 1 sensitive to paramagnetic susceptibility effects (susceptibility-weighted imaging or T2* gradient-echo), T2-weighted fluid attenuated inversion recovery, or T2-weighted imaging. Experienced trained raters used prespecified rating forms blinded to clinical details. Inter-rater reliability for ICH etiology classification was calculated (Cohen κ 0.78); any uncertainties were assessed by a senior neurovascular specialist (H.R.J., D.J.W., or T.G.).

We assessed hematoma location according to the Cerebral Haemorrhage Anatomical RaTing inStrument^15^ as lobar, deep, cerebellar, or brainstem. We further assessed hematoma volume, presence and severity of cortical superficial siderosis (cSS, disseminated if affecting >3 sulci),^16^ and markers of SVD according to the STandards for ReportIng Vascular changes on nEuroimaging criteria.^4^ The presence and distribution of CMBs were rated according to the Microbleed Anatomical Rating Scale,^17^ periventricular and deep WMHs according to the Fazekas scale,^18^ and enlarged perivascular spaces (ePVSs) according to a validated 4-point scale.^19^

We used a modified version of the CLAS-ICH classification to determine ICH etiology. We classified ICH as arteriolosclerosis in patients with a nonlobar ICH and relevant accompanying SVD (at least one of the following: ≥1 lacune; moderate or severe WMH; ≥1 deep (including brainstem) CMB; or severely ePVS in the basal ganglia [BGPVS] without lobar CMB or cSS); CAA when criteria for probable CAA based on the Boston 2.0 criteria^20^ were fulfilled; mixed location SVD when there was a mixture of both lobar and deep signs of SVD; and cryptogenic in patients without any MRI-visible signs of SVD, as previously reported.^14^ Cerebellar hemorrhages could be classified as any of the 4 neuroimaging phenotypes depending on the other imaging findings. Lobar ICH with SVD biomarkers but not meeting Boston 2.0 criteria was classified as mixed SVD. MRI SVD biomarkers were combined to give a combined SVD burden score previously used to assess total SVD burden,^12^ graded from 0 to 4 depending on the presence or absence of moderate-severe WMH (presence defined as deep Fazekas score ≥2 or periventricular score ≥3), ≥1 lacune, ≥1 microbleed, and BGPVS (graded ≥2).

### Study Outcomes

We performed a cross-sectional analysis of clinical and MRI data, investigating associations of CKD presence and severity with underlying ICH etiology, SVD burden, and individual SVD markers. The prespecified primary outcome was ICH etiological subtype. Secondary outcomes according to renal function included the presence, severity, and distribution of WMH, lacunes, CMB, and ePVS; ICH location and volume; and combined SVD burden score.

### Statistical Analysis

We described the baseline characteristics of the 2 main study groups, using mean (SD) or median (interquartile range [IQR]) depending on variable distributions and number (percentage) for categorical variables. We compared categorical variables with χ^2^ test and numerical variables with the 2-sample *t* test or Mann-Whitney *U* test as appropriate.

We investigated associations of CKD with ICH etiologies by fitting univariable and multivariable multinomial regression models, and associations of CKD with SVD biomarkers by fitting univariable and multivariable logistic regression models. ICH etiologies and SVD biomarkers were the outcome variables and CKD was the predictor variable of interest. We performed ordinal logistic regression analysis of SVD burden score against CKD. We tested the proportional odds assumption using Brant's test. Based on existing evidence, we adjusted for potential confounder variables that are plausibly associated with CKD, ICH location, and SVD type or burden. This prespecified list of variables included age, sex, hypertension, and diabetes. We included them in the multivariable models if they had a univariable association of *p* < 0.1. To adjust for covariates when comparing ICH volume between the study groups, we constructed linear regression models using log transformations of the volume owing to the skewed distribution of this variable. *p* Values of less than 0.05 were considered statistically significant.

We performed additional analyses of associations of renal function with SVD imaging markers according to the severity of CKD defined as eGFR >60, normal kidney function; eGFR 45–60, mild CKD (grade 3a); eGFR 30–45, moderate CKD (grade 3b); and eGFR ≤30, severe CKD (grades 4–5).

### Standard Protocol Approvals, Registrations, and Patient Consents

The SIGNAL registry was approved by the University College Hospital NHS Foundation Trust Governance Review Board as a continuous service evaluation of a comprehensive clinical care program (service evaluation 5-201920-SE). The Graz ICH cohort study was approved by the ethics committee of the Medical University of Graz (approval number 32-265 ex 19/20). As a retrospective cohort study including only routinely collected clinical data, the need for individual informed consent was waived.

### Data Availability

The data sets generated and analyzed during this study are available from the corresponding author on reasonable request.

## Results

**Figure 1:**
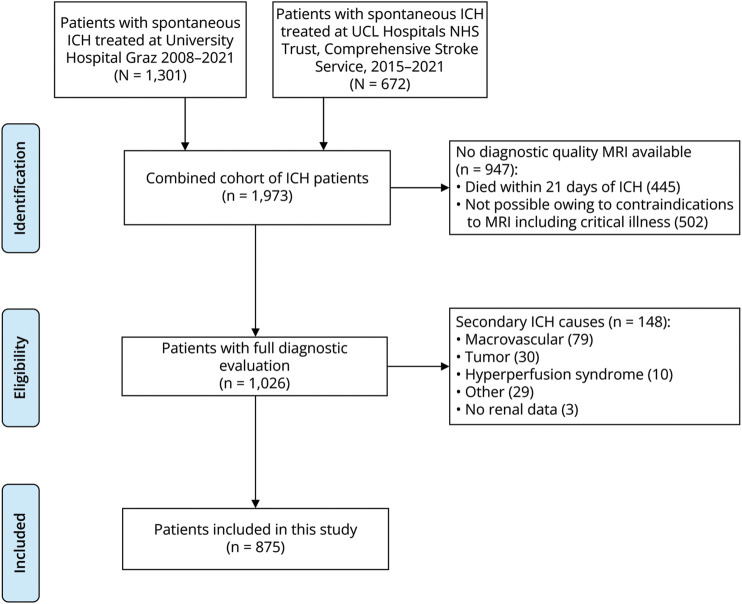
Study Flowchart of Patient Selection ICH = intracerebral hemorrhage; UCL = University College London.

A total of 875 patients met the study inclusion criteria, with mean (SD) age 66.4 (13.6) years and 57.5% male. Of total, 445 patients died within 21 days of the index ICH, and MRI was not performed in 502 patients owing to critical illness or contraindications. Of 1026 patients with ICH and available MRI, 151 were excluded (148 because of secondary ICH causes other than SVD, 3 because of missing renal data). For complete details, see the study flowchart (Figure 1). In total, 146 patients (16.7%) had CKD according to the KDIGO eGFR criteria. The group with CKD was significantly older than the group with eGFR >60, with a higher prevalence of hypertension, diabetes, previous ischemic stroke or transient ischemic attack, atrial fibrillation, and anticoagulant use. The baseline characteristics of the study participants are summarized in Table 1. Patients not included owing to missing MRI were older with mean (SD) age 71.6 (15.3) years and had a lower proportion of male patients (48.7%). Apart from a higher rate of hypertension in Graz (82.8% vs 73.5% at UCL Hospitals), there were no significant differences between the cohorts (eTable 1).

**Table 1:** Baseline Characteristics, ICH Volume, Location, and Etiology

Variable	Total	Normal eGFR (>60)	CKD	*p* Value
N	875	729 (83.3)	146 (16.7)	
Age, y, mean (SD)	67.3 (13.6)	66.4 (13.6)	71.9 (13.0)	<0.001
Sex, male, n (%)	504 (57.6)	419 (57.5)	85 (58.2)	0.868
Hypertension, n (%)	682 (78.4)	556 (76.7)	126 (86.9)	0.006
Diabetes, n (%)	160 (18.3)	110 (15.2)	50 (34.2)	<0.001
Previous IS or TIA, n (%)	122 (13.9)	88 (12.1)	34 (23.3)	<0.001
Atrial fibrillation, n (%)	119 (13.6)	83 (11.4)	36 (24.7)	<0.001
CCF, n (%)	18 (2.1)	14 (1.9)	4 (2.7)	0.524
Anticoagulant use, n (%)	106 (13.5)	77 (11.7)	29 (22.5)	0.001
eGFR, mL/min/1.73^2^, mean (SD)	75 (22)	81 (16)	45 (16)	<0.001
ICH volume, mL, median (IQR)	8.9 (3.0–22.6)	9.4 (3.3–23.1)	6.7 (2.1–16.6)	0.015
ICH location, n (%)				0.200
Deep	408 (46.6)	336 (46.1)	72 (49.3)	
Lobar	373 (42.6)	319 (43.8)	54 (37.0)	
Brainstem	32 (3.7)	23 (3.2)	9 (6.2)	
Cerebellar	62 (7.1)	51 (7.0)	11 (7.5)	
ICH etiology, n (%)				<0.001
Arteriolosclerosis	206 (23.5)	168 (23.1)	38 (26.0)	
Probable CAA	183 (20.9)	164 (22.5)	19 (13.0)	
Mixed SVD	363 (41.5)	284 (39.0)	79 (54.1)	
Cryptogenic (no SVD)	123 (14.1)	113 (15.5)	10 (6.9)	

Abbreviations: CAA = cerebral amyloid angiopathy; CCF = congestive cardiac failure; CKD = chronic kidney disease; eGFR = estimated glomerular filtration rate; ICH = intracerebral hemorrhage; IQR = interquartile range; IS = ischemic stroke; SVD = cerebral small vessel disease; TIA = transient ischemic attack.

### ICH Volume and Location

There were no significant differences in ICH location in the CKD group compared with the eGFR >60 group (*p* = 0.20). The ICH volume was larger in those with eGFR >60 than in the CKD group, median 9.4 mL (IQR 3.3–23.1) vs median 6.7 (IQR 2.1–16.6, *p* = 0.015, Table 1), but this difference was no longer significant after adjusting for age and ICH location (mean difference in log volume −0.18, 95% CI −0.41 to 0.05).

Compared with those included in the study, excluded patients had a higher median ICH volume—median (IQR) 23.3 (6.0–61.2) and median (IQR) 8.9 (3.0–22.6). There was also a higher rate of brainstem ICH (6.1% vs 3.7%) and a lower rate of lobar ICH (37.7% vs 42.6%), in the excluded group.

### Associations Between CKD and ICH Etiology

Mixed SVD and arteriolosclerosis phenotypes were more common in the CKD group than in the group with normal eGFR (54.1% vs 39.0%, and 26.0% vs 23.1%, respectively). By contrast, probable CAA and cryptogenic ICH were less common in the CKD group compared with the normal eGFR group (13.0% vs 22.5% and 6.9% vs 15.5%, respectively, *p* < 0.001 for the distribution of all etiologies). For complete details, see Table 1.

**Figure 2:**
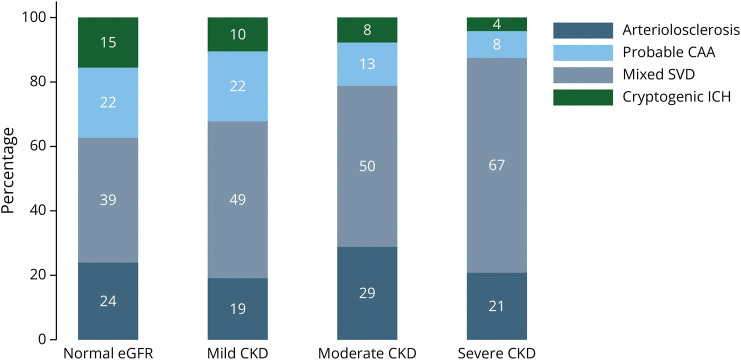
ICH Etiology According to CKD Severity CAA = cerebral amyloid angiopathy; CKD = chronic kidney disease (mild eGFR 45–60, moderate eGFR 30–45, severe ≤30); eGFR = estimated glomerular filtration rate (normal ≥60); ICH = intracerebral hemorrhage; SVD = cerebral small vessel disease.

With increasing severity of CKD, the proportion of patients with mixed SVD increased and the proportion with probable CAA and cryptogenic ICH decreased, as shown in Figure 2.

The observed association between CKD and mixed SVD was statistically significant after adjusting for age, sex, hypertension, and diabetes (relative risk ratio [RRR] 2.39, 95% CI 1.16–4.94, *p* = 0.019), as shown in Table 2. There was also a signal of an association of CKD with arteriolosclerosis as the underlying arteriopathy, after adjusting for age, sex, hypertension, and diabetes, but this was not statistically significant at the 5% level (RRR 2.04, 95% CI 0.95–4.38, *p* = 0.067).

**Table 2:** Individual Small Vessel Disease Markers According to Renal Function

Variable	Total	Normal eGFR (>60)	CKD	*p* Value
WMH, Fazekas ≥2, n (%)	488 (55.8)	377 (51.7)	111 (76.0)	<0.001
WMH, Fazekas, median (IQR)	2 (1–3)	2 (1–3)	2 (2–3)	<0.001
CMB presence, n (%)	576 (65.8)	472 (64.7)	104 (71.2)	0.131
Total CMB, median (IQR)	2 (0–8)	2 (0–8)	3 (0–9)	0.219
Deep CMB, median (IQR)	0 (0–2)	0 (0–2)	0 (0–3)	0.007
Lobar CMB, median (IQR)	1 (0–4)	1 (0–4)	1 (0–4)	0.350
Brainstem CMB, median (IQR)	0 (0–0)	0 (0–0)	0 (0–1)	0.005
Cerebellar CMB, median (IQR)	0 (0–0)	0 (0–0)	0 (0–1)	0.823
Infratentorial CMB, median (IQR)	0 (0–1)	0 (0–1)	0 (0–2)	0.078
Mixed CMB presence, n (%)	305 (34.9)	248 (34.0)	57 (39.0)	0.245
cSS presence, n (%)	115 (13.2)	103 (14.2)	12 (8.3)	0.054
Disseminated cSS, n (%)	67 (7.7%)	59 (8.1)	8 (5.6)	0.287
Lacune presence, n (%)	290 (33.1)	225 (30.9)	65 (44.5)	0.001
Total lacunes, median (IQR)	0 (0–1)	0 (0–1)	0 (0–1)	0.009
BGPVS ≥3, n (%)	289 (33.0)	230 (31.6)	59 (40.4)	0.038
CSOPVS ≥3, n (%)	405 (46.3)	345 (47.3)	60 (41.1)	0.168
Total PVS, median (IQR)	4 (3–5)	4 (3–5)	4 (3–5)	0.657
BGPVS, median (IQR)	2 (1–3)	2 (1–2)	2 (1–3)	0.003
CSOPVS, median (IQR)	2 (1–3)	2 (1–3)	2 (1–3)	0.221
Total SVD burden, median (IQR)	2 (1–3)	2 (1–3)	3 (2–4)	<0.001

Abbreviations: BGPVS = enlarged perivascular spaces in the basal ganglia; CKD = chronic kidney disease; CMBs = cerebral microbleeds; CSOPVS = enlarged perivascular spaces in the centrum semiovale; cSS = cortical superficial siderosis; eGFR = estimated glomerular filtration rate; IQR = interquartile range; SVD = cerebral small vessel disease; WMHs = white matter hyperintensities.

### Association of CKD With Individual Small Vessel Disease Markers and Total SVD Burden

In univariable analysis, there were significantly higher proportions of patients with severe WMH, number of deep CMBs, lacune presence, and BGPVS in the CKD group than those with normal renal function, as shown in Table 3. The combined SVD burden score was higher in the CKD group—median 3 (IQR 2–4) vs 2 (IQR 1–3).

**Table 3:** Multivariable Regression Models Showing Relative Risk of Each ICH Etiology and SVD Marker According to CKD and Covariates

	Predictor	aRRR	95% CI	*p* Value
ICH etiology				
Cryptogenic ICH (base outcome)				
Arteriolosclerosis	Age	1.04	1.02–1.06	<0.001
	Sex, male	0.80	0.49–1.30	0.363
	CKD	2.04	0.95–4.38	0.067
	Hypertension	1.11	0.64–1.93	0.714
	Diabetes	0.95	0.52–1.73	0.872
Probable CAA	Age	1.11	1.09–1.14	<0.001
	Sex, male	0.84	0.50–1.41	0.502
	CKD	0.88	0.37–2.08	0.775
	Hypertension	0.61	0.34–1.08	0.090
	Diabetes	0.50	0.25–1.00	0.049
Mixed SVD	Age	1.06	1.04–1.08	<0.001
	Sex, male	0.76	0.48–1.21	0.248
	CKD	2.39	1.16–4.94	0.019
	Hypertension	1.70	0.99–2.90	0.054
	Diabetes	0.73	0.41–1.29	0.281
SVD markers		**aOR**		
WMH severity (per point on the Fazekas scale)	CKD	2.03	1.45–2.83	<0.001
Deep CMB presence	CKD	1.69	1.17–2.45	0.005
Lacune presence	CKD	1.52	1.04–2.21	0.030
Severe BGPVS (≥3)	CKD	1.19	0.81–1.74	0.380
SVD burden score (per point)^a^	CKD	1.83	1.31–2.56	<0.001

Abbreviations: aOR = adjusted odds ratio; aRRR = adjusted relative risk ratio; BGPVS = enlarged perivascular spaces in the basal ganglia; CKD = chronic kidney disease; CMB = cerebral microbleed; ICH = intracerebral hemorrhage; SVD = cerebral small vessel disease; WMHs = white matter hyperintensities.

Each individual SVD marker is the outcome variable, CKD and covariates the predictors.

All models adjusted for age, sex, hypertension, and diabetes.

a To satisfy the proportional odds assumption, we needed to adjust for age group (18–50, 51–60, 61–70, 71–80, 81–100 years).

Except for BGPVS, these observed differences were all statistically significant in multivariable regression models, as shown in Table 2.

**Figure 3:**
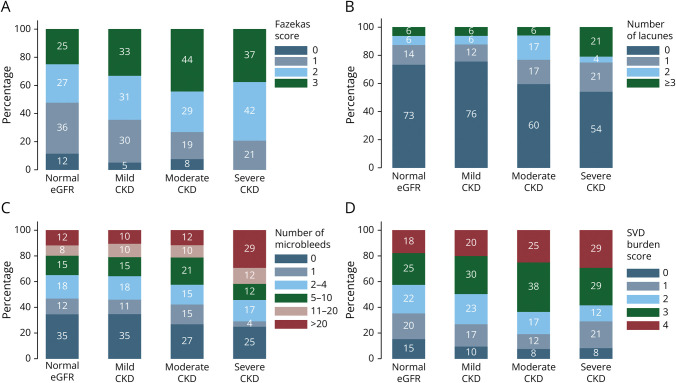
SVD Markers According to Severity of CKD for (A) WMH Severity; (B) Number of Lacunes; (C) Number of CMBs; (D) Combined SVD Burden Score CKD = chronic kidney disease (mild eGFR 45–60, moderate eGFR 30–45, severe ≤30); eGFR = estimated glomerular filtration rate (normal ≥60); SVD = cerebral small vessel disease.

The increased severity of SVD biomarkers and burden with worsening renal function is shown in Figure 3.

Because the effect was particularly marked for total number of CMBs moving from moderate to severe CKD (threshold of eGFR ≤30 mL/min), we explored this further by constructing ordinal logistic regression models using this cutoff to define severe CKD. We confirmed a significant adjusted association between CMB category (categories 0, 1, 2–5, 5–10, 10–20, and >20) and severe CKD—adjusted odds ratio 2.37 (95% CI 1.10–5.11, *p* = 0.028, eTable 2). We further investigated associations of CKD severity with SVD severity by fitting linear regression models with eGFR as a continuous outcome variable and individual SVD severity categories as predictor variables. These suggested possible nonlinear relationships of CKD severity with SVD severity, for example, with a significant estimated mean difference in eGFR of −5.9 (95% CI −10.2 to −1.6) for the CMB >20 group compared with no CMB, and nonsignificant associations with lower microbleed severities. The same was true for WMH severity and SVD burden score, but not for lacune severity and BGPVS. For complete details, see eTable 3.

**Figure 4:**
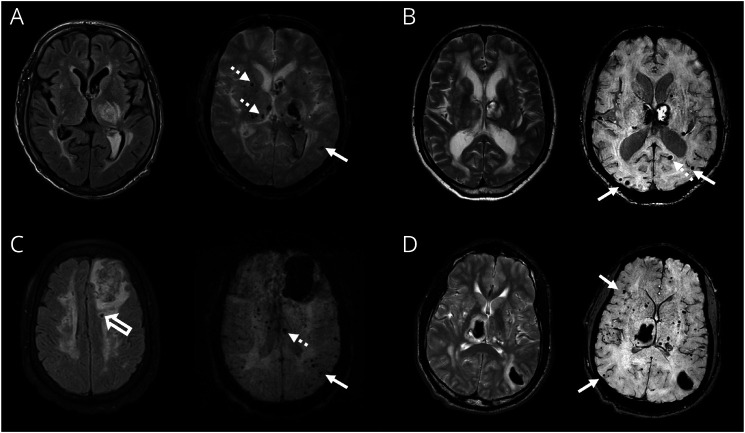
MRI Examples of Patients With Chronic Kidney Disease and Mixed Location Cerebral Small Vessel Disease (A) T2-weighted FLAIR and T2* GE MRI from a patient in their 40s with glomerulonephritis causing end-stage kidney disease requiring dialysis. There is a left thalamic ICH and mixed distribution microbleeds (lobar microbleeds, white arrows; deep microbleeds, dotted arrows). (B) T2-weighted and SWI MRI from a patient in their 60s with hypertension and stage 3a CKD (eGFR 49) with an acute left thalamic ICH, an old right thalamic hematoma, mixed pattern microbleeds, and severe WMHs. (C) T2-weighted FLAIR and GE MRI from a patient in their 40s with a history of hypertension, pre-eclampsia, and stage 3a CKD (eGFR 54) with albuminuria. There is a left frontal lobar ICH, a lacune in the left centrum semiovale (hollow arrow), severe WMH, and mixed distribution microbleeds. (D) T2-weighted and SWI MRI from a patient in their 30s with hypertension and stage 3a CKD (eGFR 56) with albuminuria. There are synchronous acute ICH in the right thalamus and left parietal lobe, moderately severe WMH, and florid mixed distribution microbleeds. CKD = chronic kidney disease; eGFR = estimated glomerular filtration rate; FLAIR = fluid attenuated inversion recovery; GE = gradient echo; ICH = intracerebral hemorrhage; SWI = susceptibility-weighted imaging; WMH = white matter hyperintensity.

The mixed pattern and severity of SVD in patients with CKD are illustrated with MRI examples in Figure 4.

## Discussion

In this large cross-sectional study of consecutive patients with ICH, MRI, and renal data, our main findings were that CKD is positively associated with overall SVD burden, a mixed SVD neuroimaging phenotype, and individual MRI markers of arteriolosclerosis. CKD was less common in CAA or cryptogenic ICH. The adjusted (independent) associations of CKD with mixed SVD ICH etiology, WMH presence and severity, lacune presence, number of deep CMBs, and total SVD burden score were stronger than with any other risk factor apart from age. The implications of our findings are, first, in the context of ICH, CKD might independently modify the severity and pattern of SVD and, second, that it may be a novel and potentially treatable risk factor. Knowledge of background history of CKD (and KDIGO eGFR stage) may help in etiology classification and risk stratification of patients admitted with acute ICH.

Although there is increasing observational evidence that renal impairment is associated with SVD,^8,21-24^ there are very few detailed studies investigating potential influence on pathogenesis in ICH populations.^9,25^ Because ICH is a severe manifestation of SVD in urgent need of effective prevention, this is an unmet research need.

An important unresolved question is whether CKD is selectively associated with certain patterns of SVD. Although there are validated criteria for CAA, and expert consensus that deep MRI markers (microbleeds, lacunes, PVS) are likely to be due to arteriolosclerosis, the interpretation of a mixed (lobar and deep) pattern of SVD is less certain, despite the consistent finding that such a pattern is common in clinical practice, accounting for around 20% of ICH associated with SVD.^5,26^ The available evidence suggests that a mixed SVD pattern more frequently might be due to more severe and widespread arteriolosclerosis^5,27^ than a true “mixture” of CAA and arteriolosclerosis. A case series of 40 brain autopsies in patients with CKD^28^ found arteriolosclerosis in 73% of specimens and just 1 specimen of CAA, potentially implicating arteriolosclerosis as the driving pathophysiologic pathway in patients with CKD and mixed SVD. Indeed, recommendations to include mixed MRI patterns in assessing ICH etiology, clinical practice, and research have recently been published.^26^ Recent cohort studies found an increased risk of recurrent ICH in the mixed SVD phenotype compared with arteriolosclerosis restricted to a deep SVD marker.^5,14^ Further investigation of the underlying mechanisms for mixed SVD is, therefore, important because it could influence ICH prevention strategies. Our novel findings suggest that CKD might be an important and independent contributory risk factor in mixed SVD. A previous small study showed an association of eGFR with deep and mixed distributions of CMB in an ischemic stroke population,^24^ consistent with our findings in a much larger ICH population.

We found increased prevalence and severity of most SVD markers related to arteriolosclerosis in patients with CKD, and the severity increased with the severity of CKD, supporting the hypothesis that CKD could be pathogenic. Previous studies of ICH populations with CKD^25,29^ and populations with advanced CKD^22,23^ have shown strong associations of CKD with markers of SVD, particularly arteriolosclerosis. In the context of these studies, our results support the hypothesis that CKD might contribute to more diffuse and severe arteriolosclerosis, possibly through mechanisms independent of hypertension and age. Recent evidence suggests that cSS is a neuroimaging marker with a high specificity for CAA.^27^ In the mixed SVD subgroup of our study, the patients with CKD had a very low rate of cSS presence (2.5% compared with 8.5% for those with normal eGFR) supporting our hypothesis that the underlying SVD in those patients is usually severe arteriolosclerosis. In addition, the rate of CAA was lower in patients with CKD, suggesting that renal function may not be as relevant to the pathogenesis of CAA, consistent with prevailing emphasis on the causal role of amyloid beta deposition in small cortical and leptomeningeal arterioles.^30^

Arteriolosclerosis is predominantly an endotheliopathy caused by dysfunction of specialized endothelial cells, particularly in the setting of cell senescence and chronic inflammation.^31^ Nitric oxide (NO)-mediated signaling pathways are particularly affected, and NO bioavailability is lower in CKD through altered expression and activity of endothelial NO synthase (eNOS).^32^ Asymmetric dimethylarginine (ADMA) accumulates in CKD^33^ and is a potent eNOS inhibitor with potential adverse effects on vascular endothelium.^34^ Pathologic SVD and accelerated cognitive impairment have been demonstrated in mouse models underexpressing eNOS,^35^ and elevated ADMA levels have been shown to be positively correlated with MRI WMH severity in humans.^36^ Another toxin associated with CKD that is potentially involved in the pathogenesis of SVD is indoxyl sulfate, a gut-derived compound normally excreted by the kidneys. It accumulates in CKD, and tissue culture models have demonstrated that it is toxic to vascular endothelium.^37^ Three rat CKD models showed increased blood-brain barrier permeability caused by increased activation of the aryl hydrocarbon receptor by indoxyl sulfate.^38^

Our findings of a graded increase in number of CMBs as eGFR decreases and reducing adjusted mean eGFR in more severe CMB categories add to previous data suggesting that CKD is potentially a strong risk factor of CMBs and hemorrhagic small vessel injury.^22,23^ In animal studies, CKD caused increased cerebral microhemorrhages in hypertensive and nonhypertensive CKD mouse models.^39^ Mechanisms proposed for CMBs in CKD mice include dysfunction of cerebral microglia and blood-brain barrier disruption. Higher transendothelial electrical resistance, a marker of endothelial dysfunction, was demonstrated in tissue culture models exposed to human CKD serum than control. This may be related to toxic uremic breakdown products, chronic inflammation, or both.^40^ These compounds could thus be targets for future research into treatments to prevent SVD progression. Examples include spherical carbon adsorbent AST-120 for intestinal chelation of indoxyl sulfate and l-arginine to counter the harmful effects of ADMA.^41^ In line with these pathophysiologic considerations, our study showed a substantially increased number on CMBs in patients with eGFR ≤30, where the concentration of these toxic substances substantially increases. However, we acknowledge that these results need to be interpreted with caution because the number of patients with severe CKD in our population was rather low.

This study has several strengths, including a large sample size, multicenter design, and a thorough validation of CKD diagnosis based on the established KDIGO definition. We are not aware of any similar studies exploring associations of definite CKD with SVD in ICH populations with such a large sample size. The multicenter design suggests that the results are applicable to a range of populations and increases the validity and generalizability of the findings.

The main limitation of our study is its cross-sectional design that can only show associations, and further prospective studies are needed to determine whether CKD is a causal or aggravating factor for arteriolosclerosis independent of shared risk factors. The cross-sectional design did not provide access to clinical outcomes, such as functional status or recurrent ICH, that are likely to be of interest to clinicians. We plan to complete a cohort study investigating these outcomes in due course. Although we adjusted for hypertension and diabetes, we did not have detailed measures of the cumulative exposure to these risk factors that could play a relevant role in these associations. Patients with CKD frequently have severe hypertension, diabetes, or both, so these will have contributed to the severe SVD found. Data on other vascular risk factors such as obesity and smoking were not available in our study cohort. Our study design does not allow us to differentiate whether shared risk factors of CKD and SVD, or the uremic endotheliopathy we have suggested, play the major role in the reported associations between CKD and the severity of SVD, although a combination of both factors is possible. Future studies should aim to adjust for shared vascular risk factors more comprehensively by including measures of comorbidity severity and cumulative exposure over time, in addition to presence. We did not have complete data on albuminuria, which means that a number of patients in the eGFR >60 group might have been diagnosed with CKD based on albuminuria. However, we defined CKD based on multiple eGFR measurements according to the established KDIGO criteria, which is more precise than previous studies on renal disease in ICH or stroke populations, which only used 1 off-eGFR measurements.^24,25,29^

In summary, our findings provide new insights suggesting that CKD might influence the pattern and severity of SVD (particularly arteriolosclerosis) independent of other established risk factors. The graded relationships between CKD severity and SVD markers and overall burden are consistent with a potential causal role, but this needs to be investigated further in longitudinal studies. If confirmed, these data could lead to new options for prevention of arteriolosclerosis and ICH; uremic endotoxins associated with CKD provide a plausible and testable hypothesis for the observed associations. Further experimental, longitudinal, or interventional studies should investigate novel treatments, for example, blocking the effects of uremic breakdown products associated with endothelial dysfunction.

## Appendix Authors

**Table:**

Name	Location	Contribution
Philip S. Nash, MBBS, MSci	UCL Stroke Research Centre, Department of Brain Repair and Rehabilitation, and Comprehensive Stroke Service, National Hospital for Neurology and Neurosurgery, University College London Hospitals NHS Trust, UCL Queen Square Institute of Neurology, London, United Kingdom	Drafting/revision of the manuscript for content, including medical writing for content; major role in the acquisition of data; study concept or design; analysis or interpretation of data
Simon Fandler-Höfler, MD, PhD	UCL Stroke Research Centre, Department of Brain Repair and Rehabilitation, UCL Queen Square Institute of Neurology, London, United Kingdom; Department of Neurology, Medical University of Graz, Austria	Drafting/revision of the manuscript for content, including medical writing for content; study concept or design; analysis or interpretation of data
Gareth Ambler, PhD	Department of Statistical Science, University College London, United Kingdom	Drafting/revision of the manuscript for content, including medical writing for content; analysis or interpretation of data
Wenpeng Zhang, MSc	UCL Stroke Research Centre, Department of Brain Repair and Rehabilitation, UCL Queen Square Institute of Neurology, London, United Kingdom	Major role in the acquisition of data
Hatice Ozkan, MPhil	UCL Stroke Research Centre, Department of Brain Repair and Rehabilitation, and Comprehensive Stroke Service, National Hospital for Neurology and Neurosurgery, University College London Hospitals NHS Trust, UCL Queen Square Institute of Neurology, London, United Kingdom	Major role in the acquisition of data
Martina Locatelli, MD	UCL Stroke Research Centre, Department of Brain Repair and Rehabilitation, UCL Queen Square Institute of Neurology, London, United Kingdom	Major role in the acquisition of data
Yang Du, MD	UCL Stroke Research Centre, Department of Brain Repair and Rehabilitation, UCL Queen Square Institute of Neurology, London, United Kingdom	Major role in the acquisition of data
Lena Obergottsberger, MD	Department of Neurology, Medical University of Graz, Austria	Major role in the acquisition of data
Gerit Wünsch, PhD	Institute for Medical Informatics, Statistics and Documentation, Medical University of Graz, Austria	Major role in the acquisition of data; analysis or interpretation of data
Hans Rolf Jäger, MD	Lysholm Department of Neuroradiology and the Neuroradiological Academic Unit, Department of Brain Repair and Rehabilitation, UCL Institute of Neurology, London, United Kingdom	Major role in the acquisition of data; analysis or interpretation of data
Christian Enzinger, MD	Department of Neurology, Medical University of Graz, Austria	Major role in the acquisition of data
David C. Wheeler, MD	Department of Renal Medicine, University College London, London, United Kingdom	Drafting/revision of the manuscript for content, including medical writing for content; study concept or design
Robert J. Simister, PhD	UCL Stroke Research Centre, Department of Brain Repair and Rehabilitation, and Comprehensive Stroke Service, National Hospital for Neurology and Neurosurgery, University College London Hospitals NHS Trust, UCL Queen Square Institute of Neurology, London, United Kingdom	Major role in the acquisition of data; study concept or design
Thomas Gattringer, MD, PhD	Department of Neurology, Medical University of Graz, Austria; Division of Neuroradiology, Vascular and Interventional Radiology, Department of Radiology, Medical University of Graz, Austria	Drafting/revision of the manuscript for content, including medical writing for content; major role in the acquisition of data
David J. Werring, PhD	UCL Stroke Research Centre, Department of Brain Repair and Rehabilitation, and Comprehensive Stroke Service, National Hospital for Neurology and Neurosurgery, University College London Hospitals NHS Trust, UCL Queen Square Institute of Neurology, London, United Kingdom	Drafting/revision of the manuscript for content, including medical writing for content; study concept or design; analysis or interpretation of data

## Glossary

ADMA: asymmetric dimethylarginine
BGPVS: ePVS in the basal ganglia
CAA: cerebral amyloid angiopathy
CKD: chronic kidney disease
CMB: cerebral microbleed
cSS: cortical superficial siderosis
eGFR: estimated glomerular filtration rate
eNOS: endothelial NO synthase
ePVS: enlarged perivascular space
ICH: intracerebral hemorrhage
IQR: interquartile range
KDIGO: Kidney Disease International: Improving Global Outcomes
NO: nitric oxide
RRR: relative risk ratio
SIGNAL: Stroke Investigation Group in North and Central London
SVD: cerebral small vessel disease
UCL: University College London
WMH: white matter hyperintensities
